# Supplementary figures and images for: Benchmarking with synthetic communities provides a baseline for virus-host inferences from Hi-C proximity linking
Source: PLoS Biol. 2025 Nov 20;23(11):e3003510. doi: 10.1371/journal.pbio.3003510 (PMC12668628; doi:10.1371/journal.pbio.3003510)

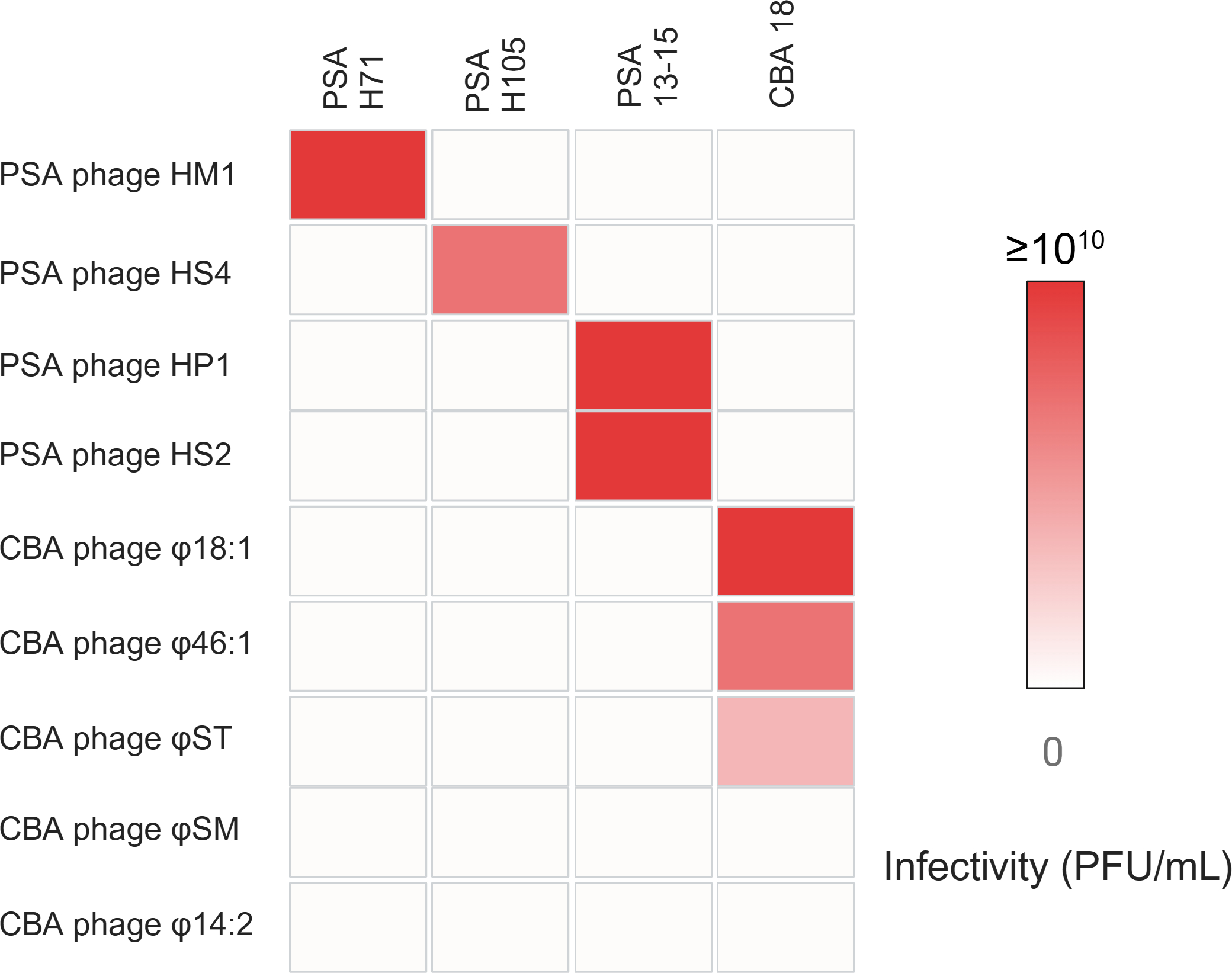

Supplement: S1 Fig — Here, the infectivity is derived from host range assays and is measured as plaque-forming unit (PFU) per mL. (PSA = Pseudoalteromonas, CBA = Cellulophaga baltica). The data underlying this figure can be found in S1 Data. (TIF) [file pbio.3003510.s001.tif]

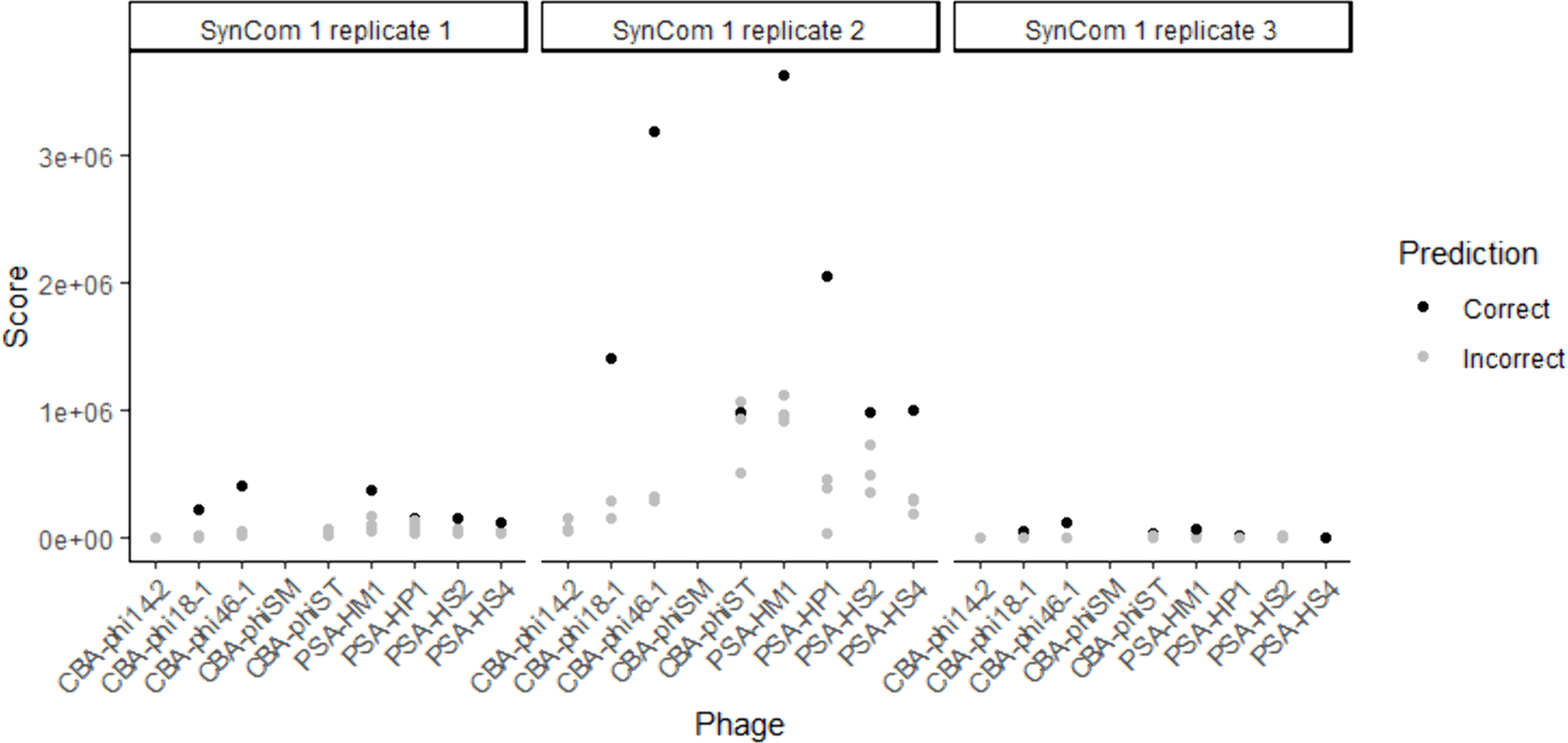

Supplement: S2 Fig — Black dots represent correct linkages and gray dots represent incorrect linkages. The data underlying this Figure can be found in S6 Table. (TIF) [file pbio.3003510.s002.tif]

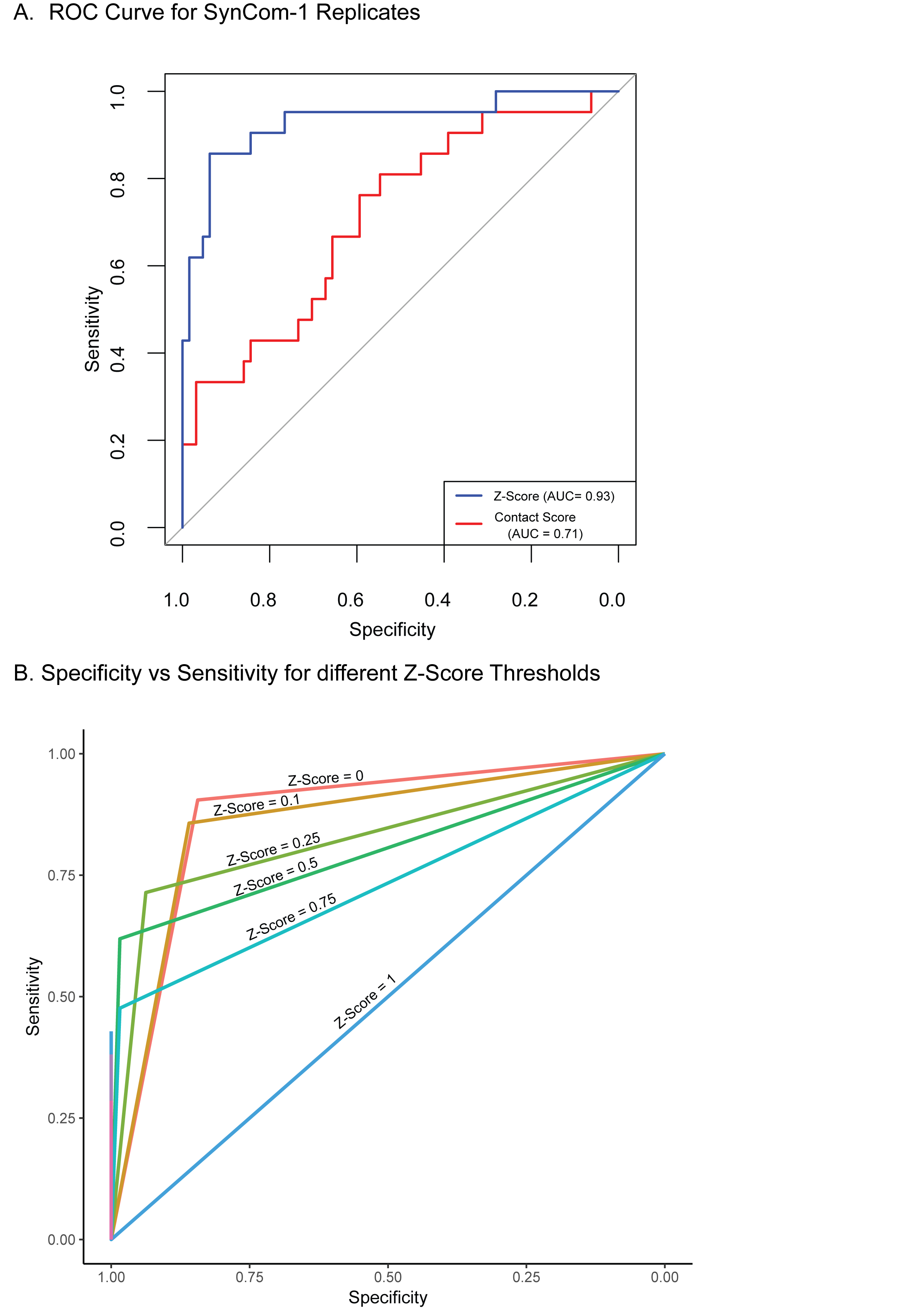

Supplement: S3 Fig — A. Receiver-operating characteristic (ROC) curve for normalized contact score (red) and Z-score (blue). B. Trade-off between sensitivity and specificity across Z-score thresholds for host-virus interaction predictions. The plot illustrates the relationship between sensitivity and specificity for six different Z-score thresholds used to filter host-virus interactions. Each line represents a distinct threshold: 0 (red), 0.1 (orange), 0.25 (green), 0.5 (light green), 0.75 (cyan), and 1 (blue). The x-axis shows specificity, and the y-axis shows sensitivity. The data underlying this figure can be found in S7 Table. (TIF) [file pbio.3003510.s003.tif]

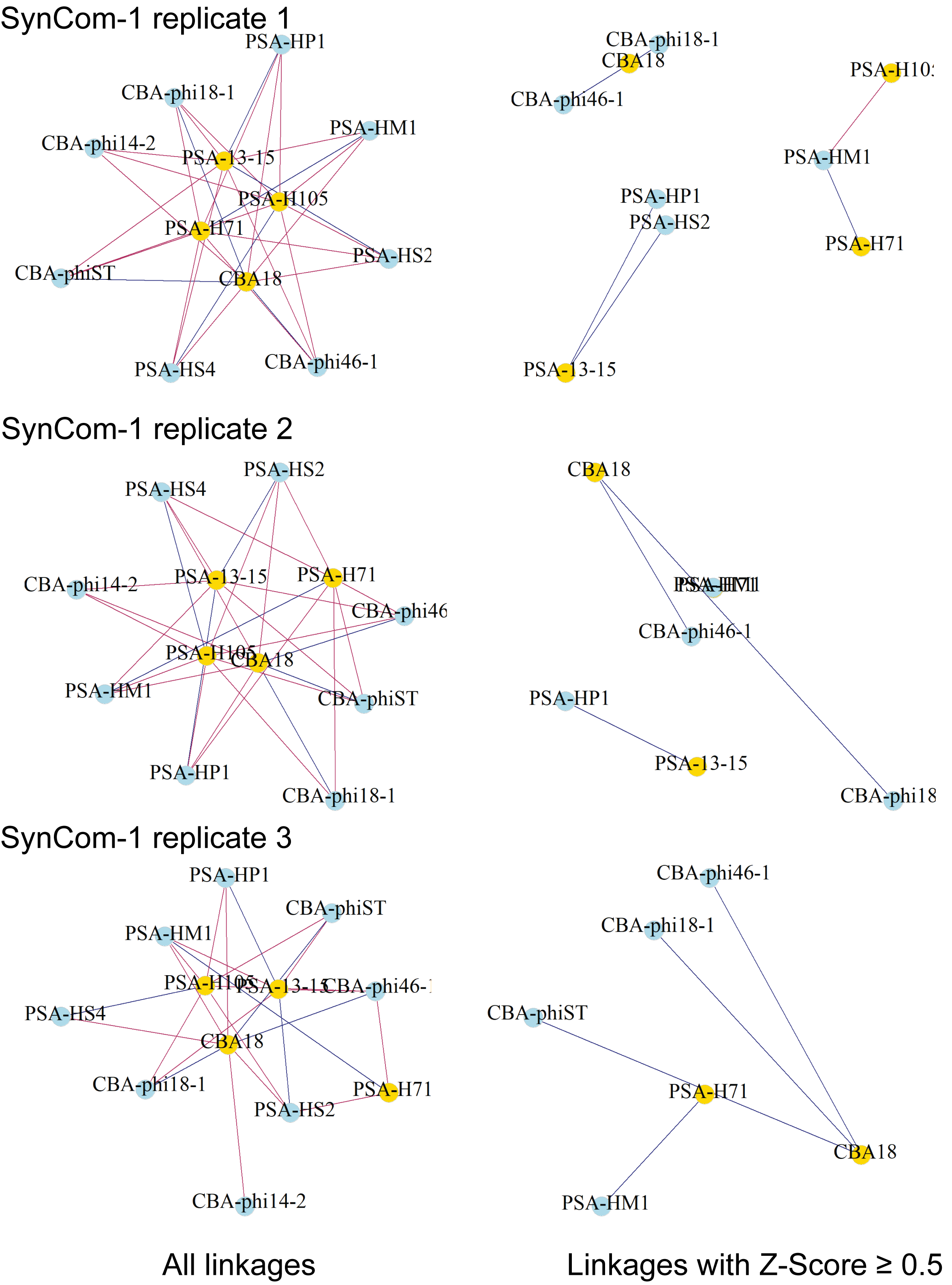

Supplement: S4 Fig — Each panel displays host-virus interaction networks for SynCom-1 replicates 1, 2, and 3. For each replicate, the left subpanel shows all detected linkages, while the right subpanel shows only statistically strong linkages with a Z-score ≥ 0.5. Nodes represent individual host or viral entities, labeled with the phage or host name. Edges between nodes indicate inferred interactions based on co-occurrence, with blue edges showing true positives and pink edges showing false positives. (TIF) [file pbio.3003510.s004.tif]

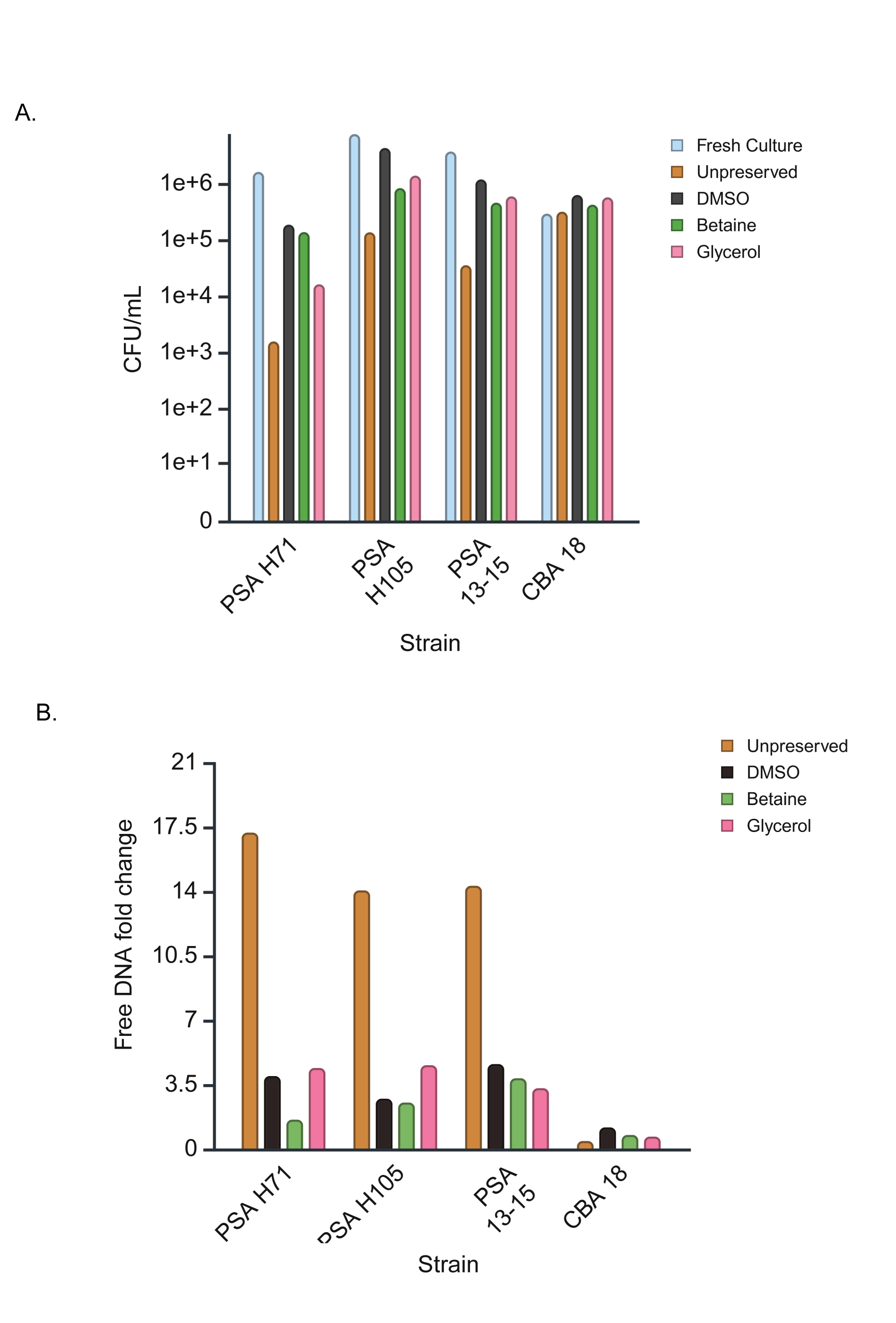

Supplement: S5 Fig — A. Viability of four bacterial strains (PSA H71, PSA H105, PSA 13–15, CBA 18) measured as colony-forming units per milliliter (CFU/mL) under different preservation conditions: Fresh Culture, Unpreserved, DMSO, Betaine, and Glycerol. B. Fold change in free DNA levels for the same strains under Unpreserved, DMSO, Betaine, and Glycerol conditions, indicating preservation-induced DNA release. The data underlying this Figure can be found in S2 Data. (TIF) [file pbio.3003510.s005.tif]

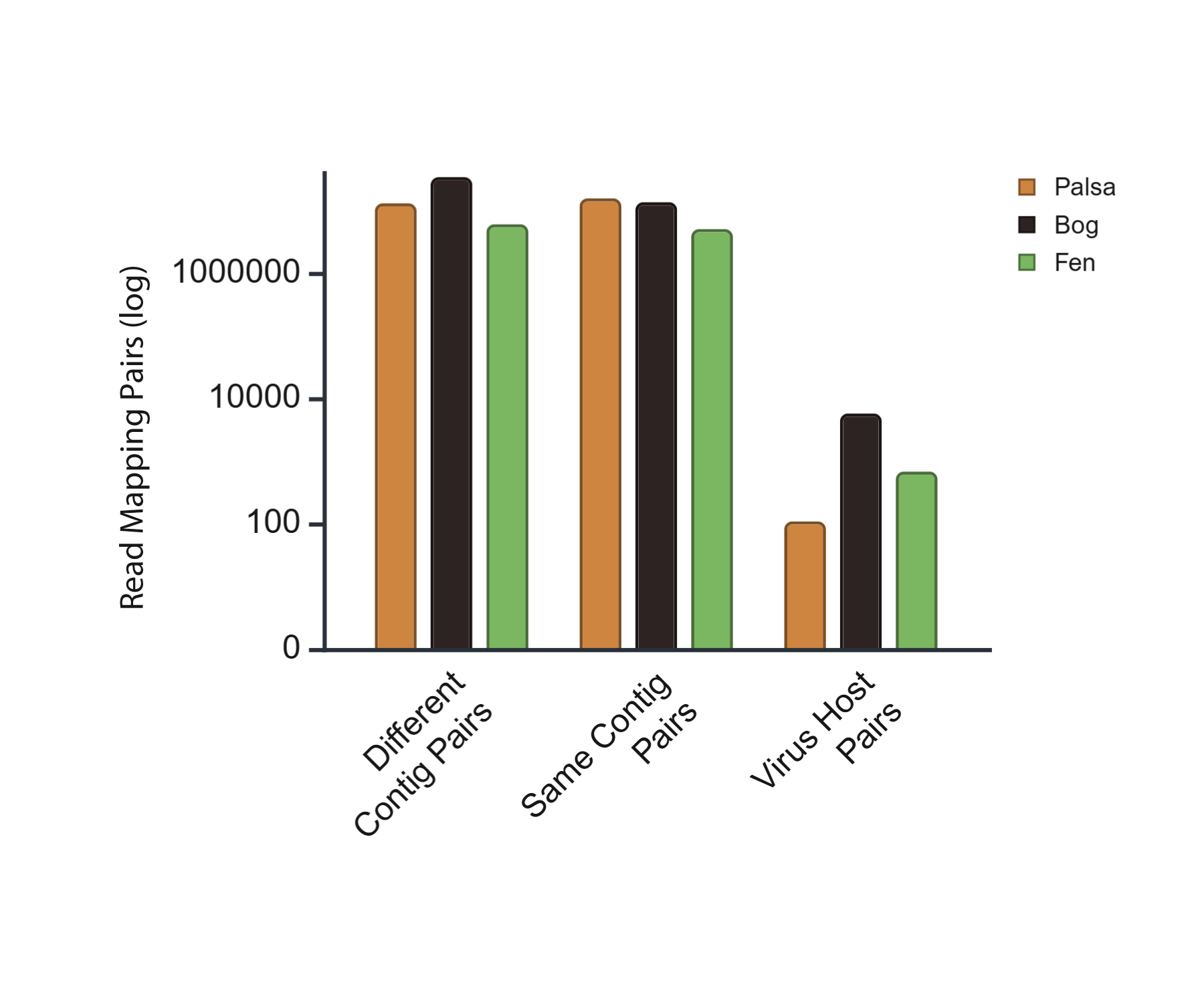

Supplement: S6 Fig — Bar graph showing the number of read mapping pairs (log scale) for different contig pairs (dark blue), same contig pairs (medium blue), and virus-host pairs (light gray) across three sample types: Palsa, Bog, and Fen. This comparison highlights the variation in genomic linkage and potential virus-host interactions across distinct peatland environments. The data underlying this figure can be found in S3 Data. (TIF) [file pbio.3003510.s006.tif]

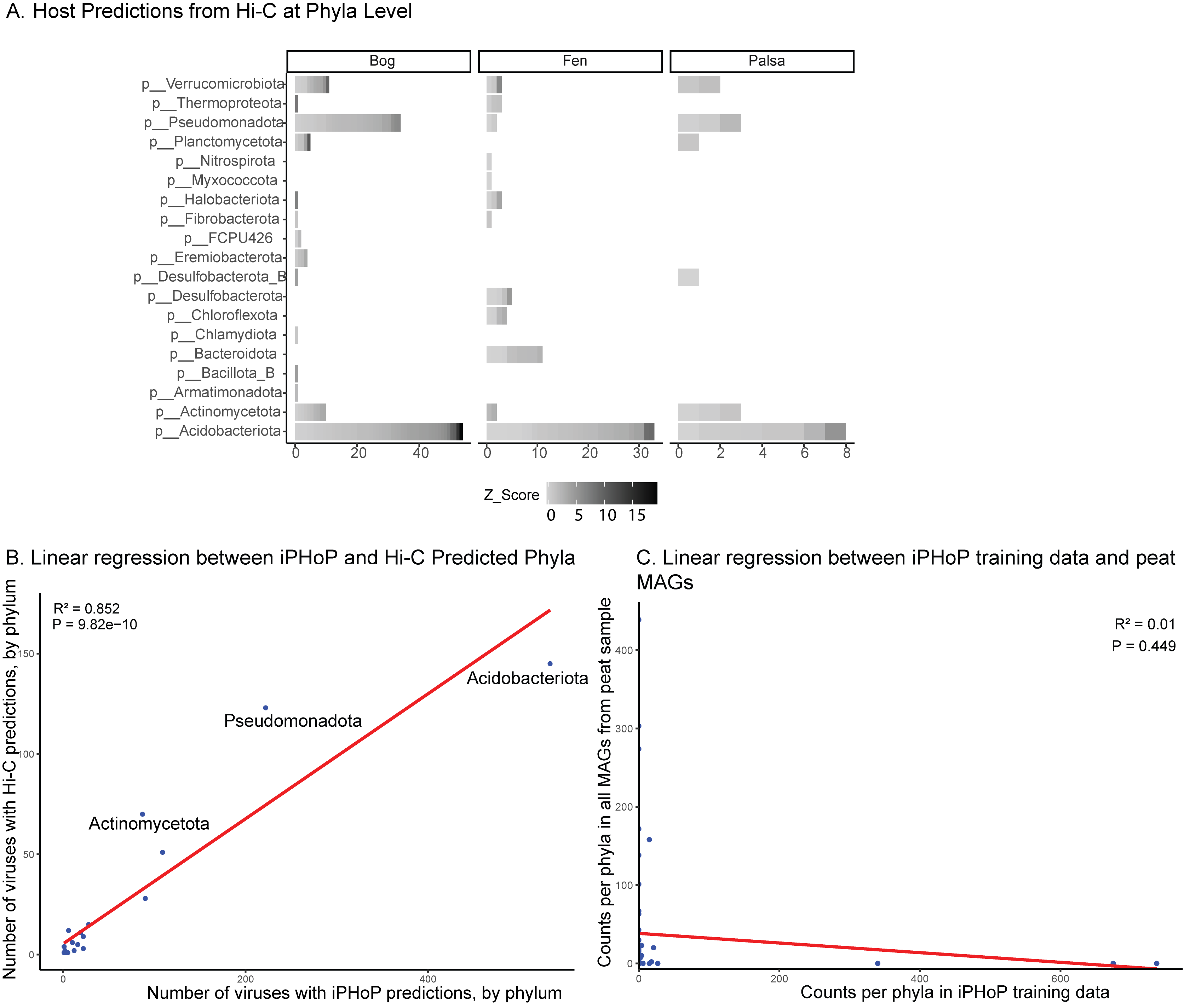

Supplement: S7 Fig — A. Host predictions (categorized at the phyla level) from Hi-C for peat samples. In each plot, the top bar shows viruses with any Hi-C linkage while the bottom bar includes only those with a Z-score ≥ 0.5. When multiple linkages were detected for a single virus, only the linkage with the highest normalized score was selected. Each phylum is represented by a different color, and the x-axis indicates the number of unique viruses. B. Taxonomic consistency between Hi-C and bioinformatic host-virus predictions. The scatter plot shows a linear regression comparing the number of phyla predicted by the bioinformatic method (iPHoP; x-axis) and the Hi-C method (y-axis). Each point represents a distinct phylum. The regression line demonstrates a strong positive correlation, with an R2 value of 0.852 and a p-value of 9.82 × 10^−10^, indicating that both methods yield similar taxonomic distributions. C. Comparison between phylum-level taxonomic counts in the MAG dataset from the soil community and the iPHoP training dataset. Each point represents a family observed in either dataset, with the x-axis showing counts from the iPHoP training data and the y-axis showing counts from Hi-C predictions from a natural community. The data underlying this Figure can be found in S12 Table. (TIF) [file pbio.3003510.s007.tif]

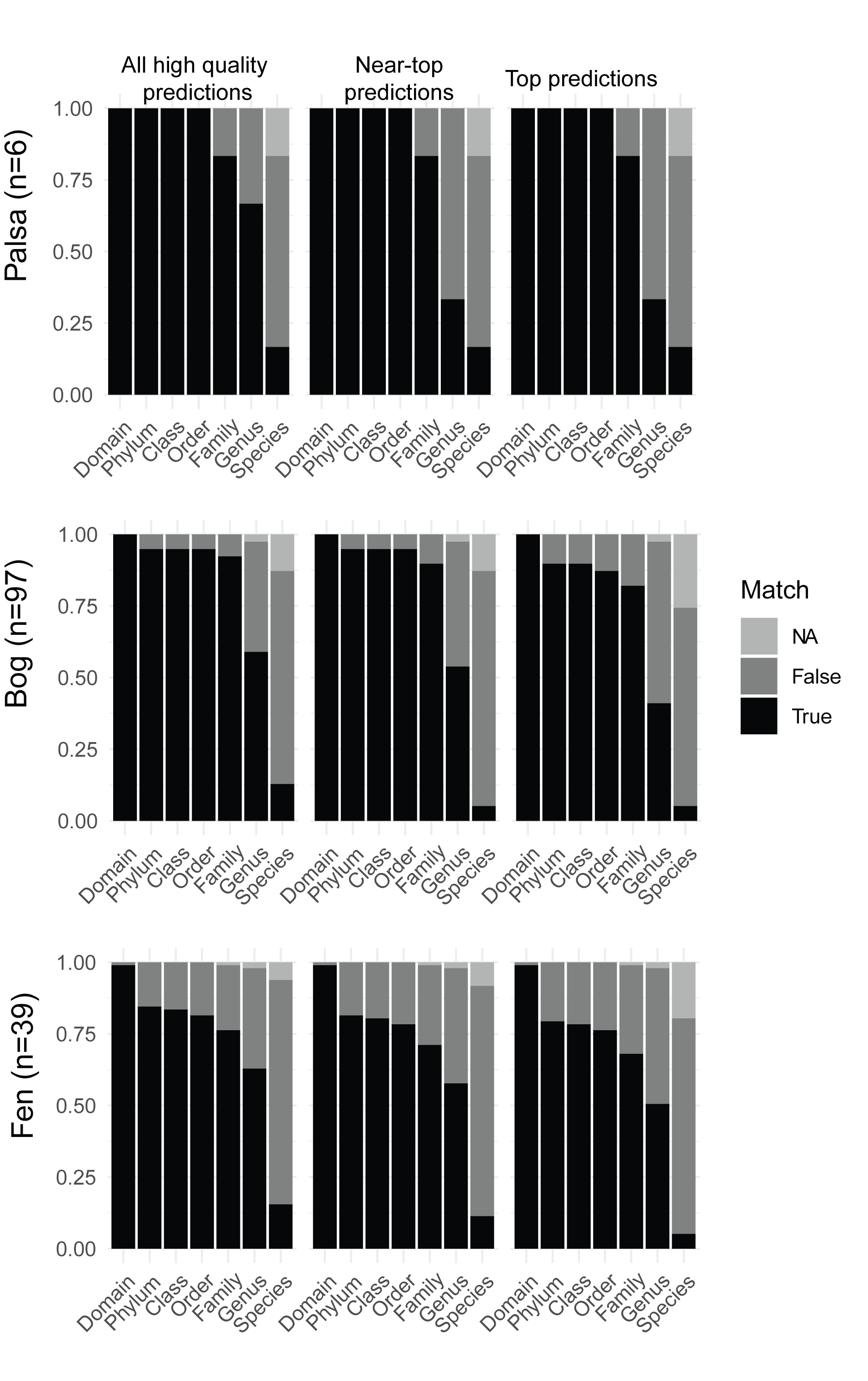

Supplement: S8 Fig — Bar plots show the agreement between host predictions from iPHoP and Hi-C across three habitats (palsa, bog, and fen). We used three different types of prediction filtering. Left column: All high-quality predictions (Hi-C Z-scores ≥ 0.5; iPHoP scores ≥ 90). Middle column: Near-top predictions (Hi-C Z-scores ≥ 0.5, all predictions with scores within 20% of the top score; iPHoP scores ≥ 90, all predictions with scores ±2 from top score). Right column: Top predictions (Hi-C Z-scores ≥ 0.5; iPHoP scores ≥ 90; only the top predictions, with multiple predictions if scores tied). The data underlying this figure can be found in S12 Table. (TIF) [file pbio.3003510.s008.tif]

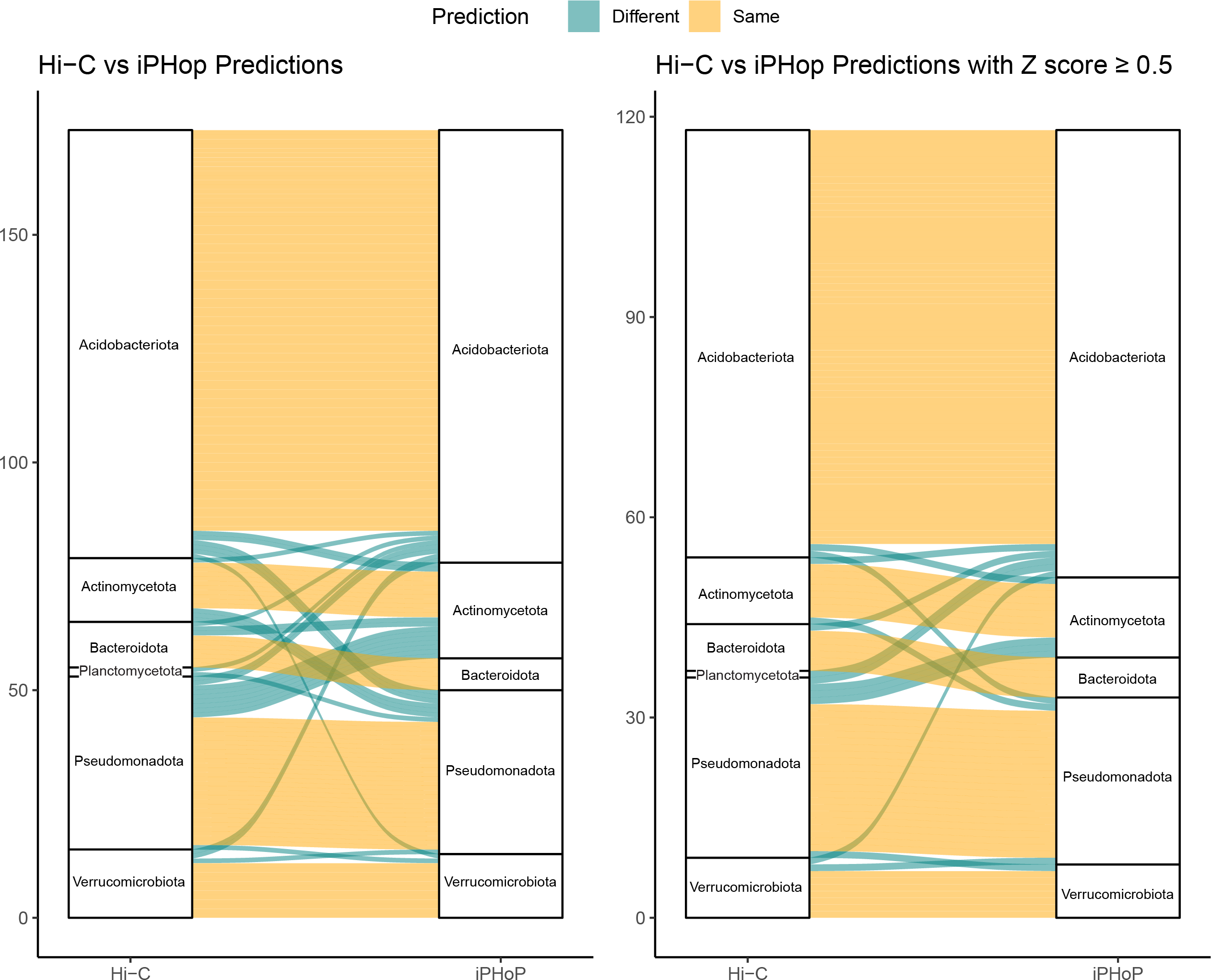

Supplement: S9 Fig — The left panel shows all phylum predictions that occurred at least five times in the dataset. The right panel shows the same, but only includes Hi-C predictions with Z score ≥ 0.5. (TIF) [file pbio.3003510.s009.tif]

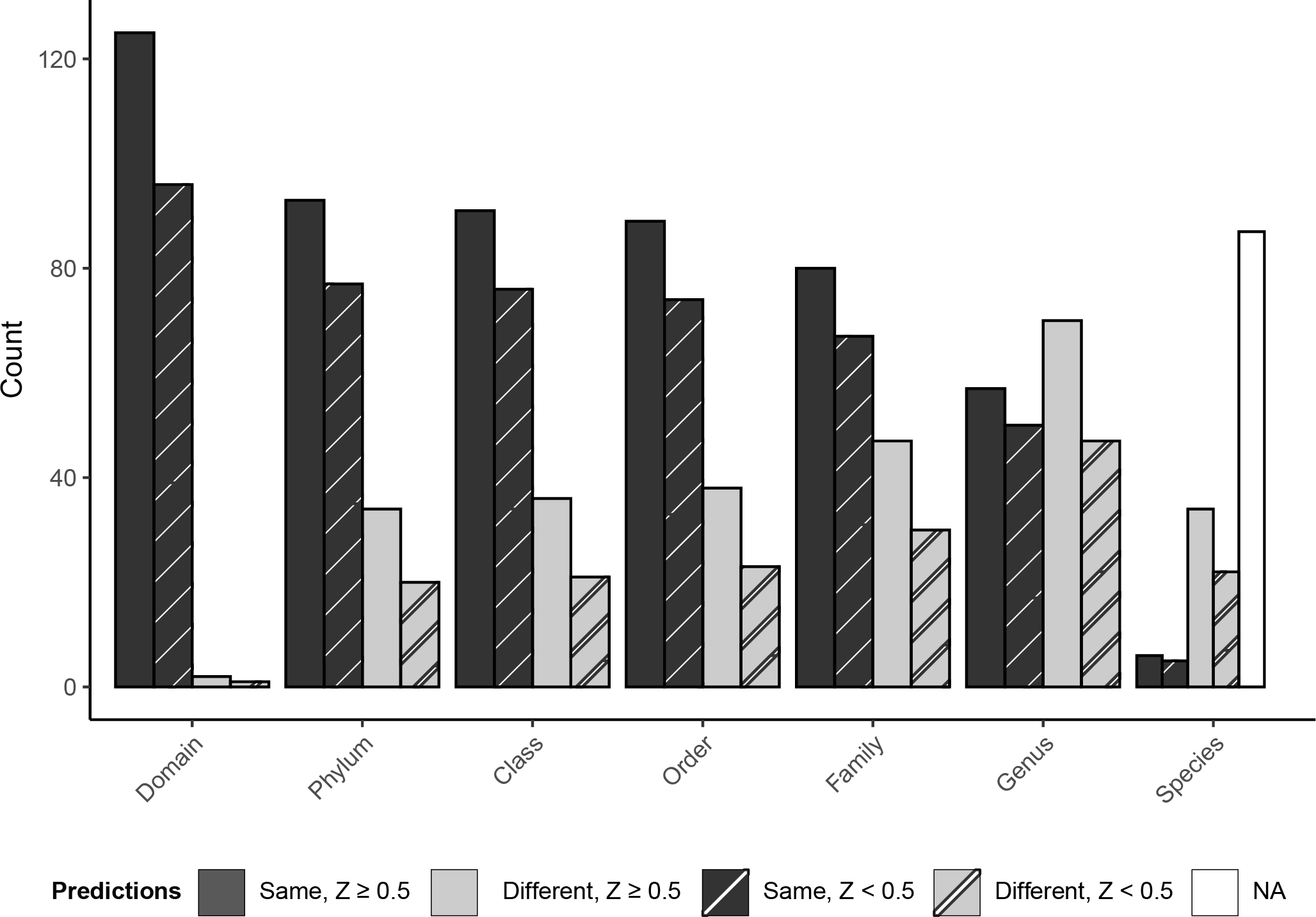

Supplement: S10 Fig — The figure summarizes the agreement between virus-host predictions from Hi-C and iPHoP across multiple taxonomic ranks (e.g., domain, phylum, class). The black bars indicate predictions that were congruent from both tools and the gray bars indicate non-congruent predictions. Solid bars and striped bars indicate predictions where the Hi-C linkage had a Z-score of 0.5 and above, and below 0.5, respectively. The data underlying this Figure can be found in S12 Table. (TIF) [file pbio.3003510.s010.tif]
