# Supplementary material for: Benchmarking with synthetic communities provides a baseline for virus-host inferences from Hi-C proximity linking
Source: PLoS Biol. 2025 Nov 20;23(11):e3003510. doi: 10.1371/journal.pbio.3003510 (PMC12668628; doi:10.1371/journal.pbio.3003510)
Supplement: Attachment — Submitted filename: Reviewer_Comments_auresp_3.docx [file pbio.3003510.s029.docx]

REVIEWER COMMENTS

## **Reviewer #1**

The manuscript systematically evaluates the performance of Hi-C technology in virus-host interaction studies, providing critical insights into its accuracy, detection limits, and sample preservation conditions through experiments involving synthetic communities (SynComs) and natural soil samples. The research design is rigorous, and the data analysis is comprehensive, offering valuable methodological references for the field of microbial ecology. However, certain aspects of the paper require further clarification and improvement to enhance its scientific rigor and readability.

Response: We thank the reviewer for recognizing the rigor of our experimental design and the value of our methodological contributions. In response to the suggestions, we have revised the manuscript to improve clarity, enhance figure quality, and deepen the analysis where appropriate.

Major comments:

1. The introduction paragraph is somewhat lengthy and scattered in focus. It is suggested to further refine the introduction to highlight the challenges of current virus-host association identification, the potential and shortcomings of the Hi-C method, and the innovation points of this study.

Response: Thank you for your feedback. In response, we have substantially revised the introduction to improve clarify, focus, and conciseness. Specially, we now clearly outline the challenges associated with current virus-host association approaches (lines 52-65), describe the potential and shortcomings of the Hi-C method (lines 83-94) and highlight the innovations introduced in our (lines 95-102). We believe these changes improve the readability of the introduction and better frame the motivation and contributions of our work.

2. The figures need to be improved. The figures have low resolution and lack visual appeal. Such as ImageGP 2 can generate high quality figures and with reproducible scripts.

Response: Thank you for your feedback. We agree that the figures, particularly the supplementary figures, required improvement. Accordingly, we have revised Figures S3-S7 to enhance resolution and visual clarity. Specifically, we re-exported all figures at higher resolution, improved color palettes for better contrast and accessibility, and standardized font sizes and styles across panels. We present Figure S5 and S6 below, to demonstrate the most dramatic changes. We also updated the main text figures (Figures 1, 2, 3, and 5) to improve interpretability and overall presentation.

To ensure reproducibility, almost all figures were generated using R-based workflows and Biorender. All associated figure-generation scripts are available on GitHub at <https://github.com/100dru/SynCom_Hi-C>. We believe these revisions significantly enhance the visual quality, clarity, and reproducibility of the figures throughout the manuscript.

**S5 Fig. Effects of preservation methods on bacterial viability and DNA integrity. A.** Viability of four bacterial strains (PSA H71, PSA H105, PSA 13-15, CBA 18) measured as colony-forming units per milliliter (CFU/mL) under different preservation conditions: Fresh Culture, Unpreserved, DMSO, Betaine, and Glycerol. **B.** Fold change in free DNA levels for the same strains under Unpreserved, DMSO, Betaine, and Glycerol conditions, indicating preservation-induced DNA release.

**S6 Fig. Log-transformed read mapping pairs across contig and virus-host relationships in three peatland sample types.** Bar graph showing the number of read mapping pairs (log scale) for different contig pairs (dark blue), same contig pairs (medium blue), and virus-host pairs (light gray) across three sample types: Palsa, Bog, and Fen. This comparison highlights the variation in genomic linkage and potential virus-host interactions across distinct peatland environments.

3. While the paper notes the low consistency between Hi-C and bioinformatics prediction tools (e.g., at the genus and species levels), it does not fully explore the potential reasons for this discrepancy (e.g., database bias or methodological limitations). Further analysis is recommended. Regarding the generalizability of the Z-score threshold, the paper mentions the need for future validation but could more specifically discuss its potential applicability in other ecosystems or sample types.

Response: Thank you for your suggestions. We agree that the limited consistency observed between Hi-C and bioinformatic host prediction tools at finer taxonomic levels warrants further exploration. One major contributing factor is likely reference database bias. Tools like iPHoP rely on curated reference genomes, which are heavily skewed towards well-studied taxa. This can lead to overprediction for organisms with abundant genomic references and underprediction of those from poorly characterized groups. In contrast, Hi-C identifies virus-host pairs through physical DNA interactions, independent of reference data. However, it is not without limitations, as there may be artifacts from sample preparation and crosslinking.

To evaluate reference database bias, we tested the hypothesis that the random forest model built into iPHoP may underperform on our soil-derived MAG dataset due to limited taxonomic overlap with the model training dataset. Specifically, we compared the 2,073 MAGs from the soil dataset (representing 48 phyla) to the phyla included in the iPHoP training dataset (for the random forest model). Only 7 phlya were shared, suggesting minimal taxonomic overlap. This was further supported by a weak correlation between the soil MAG dataset and the iPHoP training set (R² = 0.01, *p*-value = 0.449). These findings suggest that training database bias might underlie the discordance between Hi-C and bioinformatic predictions. Nonetheless, we also acknowledge that Hi-C may introduce its own methodological biases. We have added this analysis and bias explanations to the revised manuscript as follows (lines 607-616, Figure S7C):

“Discrepancies between Hi-C and bioinformatic predictions may stem from database biased training that iPHoP uses (e.g., favoring well-studied taxa) or Hi-C methodological artifacts (e.g., spurious contacts introduced during cross-linking or processing, differential membrane resistance to cross-linking chemistries, interference from relic DNA). While the latter are challenging to address experimentally (often requiring costly or currently limited single-cell approaches), we explored whether iPHoP’s random forest model was trained on host genomes taxonomically representative of those found in our soil samples. Of the 48 phyla identified in our soil MAG dataset, only 7 were present in the iPHoP training set, yielding a weak correlation (R² = 0.01, p = 0.449; **S7C Fig**). This limited overlap suggests that training dataset database bias is at least one key factor underpinning the discordance between Hi-C and bioinformatic predictions.”

**Figure S7C.** **Comparison between phylum-level taxonomic counts in the MAG dataset from the soil community and the iPHoP training dataset.** Each point represents a family observed in either dataset, with the x-axis showing counts from the iPHoP training data and the y-axis showing counts from Hi-C predictions from a natural community.

Regarding cross-environment generalizability of the Z-score threshold and the reviewer’s suggestion to speculate on this more in the manuscript, we have now added the following text to this effect (lines 635-649).

“Assuming Hi-C proximity ligation becomes more broadly used, it becomes important to assess whether a Z-score threshold of ≥ 0.5 is broadly generalizable. While our SynCom data indicates that this threshold effectively removes spurious linkages, these results are based on controlled experimental conditions using marine phage-host experimental model systems. The foundational step in Hi-C proximity ligation is the use of a generalizable cross-linking molecular treatment to capture physically proximal DNA fragments, making the method theoretically applicable across diverse environments. However, natural systems may introduce variability that affects the reliability of this threshold. For example, soil environments may contain taxa with unique cell membrane structures, high concentrations of humic substances, and/or particle-associated relic DNA. All of these could complicate cross-linking efficacy or downstream molecular biology steps, whereas these issues might be drastically reduced in marine environments.”

4. Descriptions for some figures (e.g., Figures S5-S8) are relatively brief. It is recommended to explain their key information in more detail in the main text. The Venn diagram in Figure 5 could include specific numerical values or percentages to more intuitively illustrate the differences in consistency between methods. A better Venn diagram webserver EVenn is recommended to show better and high-quality results.

Response: Thank you for your comments. We have significantly expanded the figure legends for all supplementary figures, with particular attention to those for original Figures S5–S8, which are now Figures S6 – S7, S9, S10. Additionally, we have incorporated more details descriptions of the key findings from these figures into the main text (Figure S6: Lines 546-547; Figure S7A: Lines 557-559; Figure S9: Lines 592-595, Figure S10: Lines 596-599).

Regarding Figure 5A, we have revised the Venn diagram to include specific numerical values for each region, and regenerated the figure using EVenn to enhance visual quality and clarity.

**Figure 5A. Comparison of virus-host prediction from Hi-C and *in silico* tools. A.** Euler plot showing the overlap of viruses with host predictions obtained from the experimental Hi-C linkage approach, or one of two *in silico* tools (iPHoP and VirMatcher) that use different probabilistic models to aggregate output of various sequence-based features to create host prediction scores.

5. If possible, it is recommended to include examples of raw data (e.g., alignment results of partial Hi-C reads) in the supplementary materials to enhance methodological transparency.

Response: Thank you for your suggestion. As there is currently no standardized format for reporting raw data in Hi-C based virus-host studies, we aimed to be as transparent as possible. To support reproducibility, we have provided the full table of normalized contact counts and Z-scores (S9 Table). In further enhance methodological transparency, we have made example Hi-C alignment data (in the form of SAM files) available in our study’s GitHub repository (<https://github.com/100dru/SynCom_Hi-C>). The GitHub also includes methodological instructions (reviewing all steps of the workflow), allowing others to examine and reproduce our read-mapping and linkage analyses.

6. A network connection with host and virus is also recommended, such as iNAP 2 (10.1002/imt2.235) or ggClusterNet 2 are good tools for network analysis.

Response: Thank you for your suggestion. In response, we performed a network analysis using of host-virus interactions within our synthetic community (SynCom-1), visualized using the igraph package (https://r.igraph.org/) across three technical replicates. Each network is presented in two panels: one showing all detected linkages and another displaying only those with a Z-score ≥ 0.5, indicating statistically stronger interactions.

This analysis revealed consistent and reproducible host-virus interaction patterns across replicates. Notably, because virus-host linkages were known, these analyses revealed that many spurious linkages were apparent prior to Z-score filtering, which highlights the value of applying a threshold to reduce noise and enhance biological signal.

We integrated these findings into our study, with new Figure S4 (see below), with corresponding updates in the main text (lines 386-389):

“Moreover, this Z-score-based threshold showed consistent performance across all replicates, as further supported by network analyses (**S4 Fig**), which showed a substantial reduction in false positives while retaining true positives (pink and blue edges, respectively).”

**S4 Fig. Network analysis of host-virus interactions in SynCom-1 across three technical replicates.** Each panel displays host-virus interaction networks for SynCom-1 replicates 1, 2, and 3. For each replicate, the left panel shows all detected linkages, while the right panel shows only statistically strong linkages with a Z-score ≥ 0.5. Nodes represent individual host or viral entities, labeled with the phage (blue) or host (yellow) name. Edges between nodes indicate inferred interactions based on co-occurrence, with blue edges showing true positives and pink edges showing false positives.

Minor comments:

7. Which kinds of Hi-C you used. Recently published GutHi-C in iMeta. What the similar or different with GutHi-C, please compare and explain in the discussion paragraph.

Response: Thank you for your comment. In this study, we used a traditional Hi-C approach that has been widely applied in recent studies to improve genome binning (as reviewed in (1)) and, as in our case, to associate viruses with their microbial hosts. Specifically, we employed the Phase Genomics ProxiMeta kit for Hi-C library preparation, which, to our knowledge, is the only commercially available kit designed for microbial Hi-C applications. For downstream processing, we used MetaCC to normalize the Hi-C linkage data, which formed the basis for the virus-host linkage analyses presented in the manuscript.

GutHi-C introduces optimized Hi-C sample processing steps, specifically for human and gut microbiome studies. While the study did not benchmark Hi-C accuracy via any synthetic community experiments, it reported improvements in alignment rate (unique alignment rate), valid pair ratios, and effective data yield as compared to the Proximeta kit. However, GutHi-C focused exclusively on recovering high-quality microbial genomes and did not evaluate virus-host linkages.

Given our study’s focus on benchmarking virus-host linkages inferred through Hi-C, we chose to limit comparisons to studies that directly involve Hi-C derived virus-host linkages. As GutHi-C did not address this aspect, we did not include GutHi-C in the introduction, but we mention it in the results (lines 646-649):

“Towards this, researchers working on the gut microbiome have already begun adapting key steps in the Phase Genomics ProxiMeta Hi-C kit protocol, such as increasing the volume of the crosslinking reaction and substituting restriction enzymes (e.g, using Dpn II instead of Sau3AI and MluCI) to optimize performance for their specific sample types (2).”

8. The terms such as "bacteriophage" and "phage" should be unified in the text to avoid confusion.

Response: Thank you for this suggestion. We revised the text to ensure consistent use of the term “phage” throughout the manuscript.

9. Some sentences are overly long and structurally complex (e.g., the first paragraph of the Introduction). Splitting or simplifying them is recommended to improve readability.

Response: Thank you for your comment. We have significantly revised the entire introduction to improve clarity by simplifying overly long and complex sentences.

10. The paper mentions failures in Hi-C library preparation for certain samples (e.g., glycerol-treated samples) but does not explore potential reasons in depth. It is recommended to supplement related analyses or hypotheses, such as whether this is due to reagent compatibility or sample processing steps. For the weaker Hi-C signals observed in natural soil samples, potential biological or technical factors (e.g., soil complexity or DNA extraction efficiency) should be discussed.

Response: Thank you for your comment. All glycerol-treated samples failed to yield a significant number of Illumina reads (< 1M reads), preventing downstream analysis. While the exact cause remains unclear, we suspect reagent incompatibility at some stage of the Hi-C workflow, as the reviewer suggests. We evaluated DNA input quantity, Hi-C library quality, and sequencing library quantity, and did not observe notable differences compared to other cryo-preservative, suggesting the failure might be due to glycerol incompatibility. We have updated the results section to reflect this interpretation (lines 428–431):

“Only DMSO (3 replicates) and betaine (2 replicates) could successfully be Hi-C library prepared, while none of the 3 glycerol replicates were successful possibly due to reagent incompatibility (see Materials and Methods).”

Regarding weaker Hi-C signals observed in the natural soil samples, biological, physicochemical and/or technical factors may be involved. For example, low infection frequencies *in situ* could result in fewer physically linked virus-host pairs. In addition, humic substances commonly found in soil may act as inhibitors for DNA-based molecular methods such as PCR and restriction digestion (3). Both are critical steps in the Hi-C protocol so these inhibitory effects may cause a weaker Hi-C signal (3). We have added this discussion to the main text (lines 535-538).

“These findings demonstrate that while spike-ins are ideal for quantitative work (4–7), optimizing spike-in inocula for field samples, particularity in soils, presents numerous challenges such as presence of humic substances that may inhibit DNA-based molecular reactions.”

11.      Labels and annotations in Figures 2 and 3 could be more explicit, such as directly marking "true positives" and "false positives" with specific examples.

Response: Thank you for your feedback. Figures 2 and 3 shows exactly all the interactions present in the SynCom-1 with different treatments. True positives are indicated with the color black while false positives are shown with the color gray. We also highlight representative examples of both true and false positives within the main text. For Figure 2, we write (lines 333-338):

“Expected linkages included all seven known interactions identified in prior single phage-host infection assays (**Fig 2, S1 Fig**), with 100% true positive detection (sensitivity) across three replicates (**S6 Table, S7 Table**). Unexpected linkages were for phage-bacteria pairs not known to infect that we included to represent negative controls (**Fig 2, S1 Fig**). For example, φ14:2 lacked a corresponding host in SynCom-1, and yet it showed linkages with multiple bacterial strains, and this finding persisted even across replicates (**Fig 2B**).”

For Figure 3, we write (lines 441-447):

“For those replicates that led to sequencing libraries and data, we examined the specific linkages between PSA 13-15 (to HS2), PSA H105 (to HS4), and CBA 18 (to φST) in the cryopreserved samples to determine if they improved. Despite having similar total linkages (**S5 Table**), there was no consistent improvement in the cryopreserved samples for these linkages. The only (slight) improvement being DMSO-treated SynCom-1 samples where PSA H105 - HS4 linkages were detected above Z-score threshold in two out of three replicates.”

12.      It is recommended to use a ROC curve as a supplementary illustration to the Z-score distribution plot to enhance the intuitiveness of the evaluation criteria.

Response: Thank you for your suggestion. In our initial submission, we included a plot of Area Under the Curve (AUC) versus Z-score threshold, which serves as a proxy for ROC curve behavior across varying thresholds for SynComs-1 (Figure S3A). To further enhance interpretability (and to address additional comments), we have now added: a table of individual AUC values for each SynCom replicate (Supporting Information), and a plot of sensitivity versus specificity across different Z-score thresholds (Figure S3B). We believe these additions provide a move intuitive and comprehensive view of how Z-score thresholds were evaluated and support our rationale for selecting the final cutoff.

The manuscript makes a significant methodological contribution, providing practical technical guidance and benchmark data for virus-host interaction research. With the aforementioned improvements, its scientific value and reader-friendliness can be further enhanced. It is recommended for a major revisions.

Response: We thank the reviewer for their thoughtful evaluation and recognition of the study’s methodological contributions. In response to the recommendation for major revisions, we made substantial improvements throughout the manuscript—including adding network analysis, more extensively comparing Hi-C with bioinformatic tools, expanding the discussion on Z-score rationale, and enhancing overall clarity. We believe these revisions have significantly strengthened the manuscript and improved its accessibility.

## **Reviewer #2**

13. Although the study question is quite actual and the study design and methodology are overall valid, the significance and novelty of the results presented do not appear to be corresponding to the level of the journal. In other regards, below are some specific comments:

Response: We appreciate the reviewer’s perspective and acknowledge that we may not have fully emphasized that importance of our findings in the initial submission. However, we feel strongly that this study is both novel and impactful, and merits publication in *PLoS Biology*.

At present, there is a proliferation of “blind” uses of Hi-C for inferring virus-host linkages in the literature. Our literature review (**S8 Table**) reveals at least ten published studies that use Hi-C for this purpose without benchmarking or validation of accuracy. Table S8 summarizes the methodologies and filtering criteria used in each study. Many studies interpret raw Hi-C signals (chimeric reads that map to a virus and a host) as indicative of a virus-host association, whereas some apply minimal filtering (e.g., removing linkages supported by fewer than something between two and ten reads) where the filters are arbitrary, non-standardized, and lack empirical justification for the cutoffs chosen. As a result, the accuracy and reliability of the reported linkages remain unknown.

Our study systematically optimizes and benchmarks Hi-C for virus-host inference using SynComs with known interactions. We show that, on average, 75% of virus-host linkages were incorrect when no filtering was applied, which calls into question interpretations made in prior studies.

Of the 10 published studies that used Hi-C inferred virus-host linkages, eight lacked a virus-host linkage table with a scoring metric or confidence assessment (**S8 Table**). The lack of standardized and accessible data, particularly the absence of raw linkage tables and accompanying metadata, makes comprehensive cross-study comparisons infeasible without extensive reprocessing of raw data from each study. Among the remaining studies, only one (Hwang et al 2023, Nature Microbiology) provided a table of virus-host linkage information in a format compatible for downstream analyses. We reanalyzed the publicly available dataset from this paper and found that 70% of their reported linkages fall below our proposed cutoff. Notably, the major finding in that study that reported cross-domain virus-host associations (e.g., a bacterial virus interacting with archaeal host) has never been observed in a laboratory setting and is not supported using our benchmarked Z-score filtering method.

Other Hi-C based studies, such as those in groundwater (8) and soil (9) environments have reported virus-host linkages that span phyla, associations that have never been demonstrated under laboratory conditions. Although we were unable to perform even a preliminary re-analysis of these, we suspect that, had data been accessible, many of these reported linkages would not withstand scrutiny using our empirically benchmarked Z-score thresholding approach.

Our results demonstrate that spurious virus-host linkages can be reduced by implementing empirically derived thresholds, in contrast to the naïve applications used in prior published studies. Our proposed threshold is not intended to be universal, as optimal cutoffs may vary across environments and experimental conditions. Instead, it offers a framework for tailoring and validating Hi-C methods, highlighting limitations in previous (high-profile) studies, and promoting more rigorous, context-specific standards.

Given the broad appeal of the method and its extensive use in microbial ecology, our benchmarking, proposed guidelines, and accompanying case study are critical for elevating the field towards rigorous, reproducible, and accurate science. To this extent, we have developed a shared, community-driven resource within the iVirus platform that would allow researchers to evaluate and interpret the virus-host linkages from other publications and different ecosystems ([www.ivirus.us/proximity-ligation](http://www.ivirus.us/proximity-ligation)).

Abstract:

14. "Microbiomes influence diverse ecosystems, but viruses increasingly appear to impose key constraints." - why "but" rather than "and" ?

Response: Thank you for your correction. We revised the sentence to use “and” instead of “but”.

15. Typo: "virushost"

Response: Thank you for the correction. We fixed the typing mistake.

16. "optimized protocols" - Worthy specifying if the optimization refers to the wet-lab part or rather the data analysis alone.

Response: Thank you for your correction. We clarified the sentences in the abstract to refer to “optimized bioinformatic protocols” (lines 24, 30).

17. "Hi-C inferred virus-linkages with in silico bioinformatic predictions" - Isn't the former a subset of the latter? Maybe the latter was supposed to be more specific (i.e. sequence-based predictions).

Response: Thank you for your suggestion. We have revised the sentence for clarity (lines 29-32):

“Applying optimized protocols to natural soil samples, we compared virus-host linkages inferred from proximity-ligated Hi-C with predictions generated by *in silico* homology-based and machine learning-based bioinformatic approaches.”

Intro

18. Line 44: "including humans where they can influence cravings" - It is not clear why the term "cravings" is listed #1 among the roles of human gut microbiomes and what that means.

Response: Thank you for the correction. We have significant revised the introduction. The sentence now reads (lines 44-46):

“Beyond their contributions to environmental processes, microbes support plant productivity, affect animal development, and influence human health, behavior, and diseases susceptibility.”

19. Line 55: missing punctuation "oceans (9-11) lakes".

Response: Thank you for the correction. We have significantly modified the introduction, so the original sentence has changed. However, we have ensured the appropriate punctuation is now included in the list of diverse ecosystems.

20. Line 90: "Hi-C method remains largely unoptimized" - Please specify if you are referring to the wet-lab part or bioinformatics or both here.

Response: Thank you for the comment. We revised the manuscript to clarify that it is the bioinformatic analyses that remain largely unoptimized (lines 87-88).

21. Line 101: Please specify what unites the bacterial taxa selected as SynComs' components in terms of their environmental niche. Are they all marine? This is worthy being mentioned in the Abstract as well.

Response: Thank you for the question. All the bacteria in the SynComs are marine isolates. We have revised the manuscript to clarify this point by explicitly stating their marine origin in both the abstract (line 23) and introduction (line 95).

22. Also please explain why the niches were different for the SynComs and the real-life communities - marine vs. soil. Could it have served as a bias - i.e. the principles that worked for marine microbes being possible different from the soil ones?

Response: Thank you for your comment. The core principle of the Hi-C method (capturing physical proximity between phage and host DNA within intact cells) would seemingly be environment-independent but could be impacted by ecosystem-specific variation in inhibitors of molecular biology. For example, we expect that peatland active layer soils, with their extensive humics, could inhibit key molecular biology steps of the Hi-C sample processing protocols. We selected marine bacteria and phages for SynComs because their interactions were well characterized and supported by extensive host range data. This allowed us to construct a controlled and reproducible synthetic community, which was critical for benchmarking the Hi-C method. Given the findings that marine biomass levels might often fall below the detection limit our SynCom experiments determined for the assays, we pivoted to soil samples as they have more microbial biomass.

We acknowledge that environmental context (marine vs. soil) could introduce potential bias. For example, soil communities tend to have greater complexity and thus may have more virus-host linkages than marine. Moreover, a higher level of relic DNA could potentially contribute to noisier Hi-C linkage signals and an increased risk of false positives by having unspecified linkages. The humic substances present in the soil may also cause molecular biology steps and have a different signal intensity compared to marine. This underscores the importance of applying stringent filtering criteria, such as our Z-score approach, to distinguish true interactions from background noise. We have added a discussion of this potential bias in the manuscript (line 518-522):

“Furthermore, since the SynComs originate from a different environmental system (marine), they are taxonomically distinct from the peat community and serve as effective internal controls for thresholding. While environmental differences (e.g. chemistry) may introduce some bias, such as varying levels of complexity or relic DNA, the core Hi-C principle of capturing physical proximity within intact cells remains consistent across ecosystems.”

Materials and Methods

23 line 232: "we retained only the top host prediction for each virus" - Were the cases when multiple hosts scored (almost) equally high in comparison with the rest treated specially? If not, then is not the approach unstable in defining the host in such situations, affecting the results and conclusions?

Response: Thank you for your insightful comment. In our Hi-C experiments, many viruses showed linkages to multiple potential hosts, even after Z-score threshold filtering. For simplicity, we focused on the top-scoring prediction (or the first among tied top scores). You are right that our originally submitted manuscript left it uncertain how similarly high-scoring candidates might impact host assignments.

To evaluate this in revision, we performed new analyses comparing Hi-C versus iPHoP (not VirMatcher due to the many more predictions iPHoP made) host predictions across three stringency levels: 1) all high-quality predictions, 2) near-top predictions, and 3) top predictions only (including ties; see methodological details in Figure S8 legend for details). Results showed no significant differences between the three approaches (pairwise Fisher’s tests) at any taxonomic rank and stringency. While matching predictions slightly decreased when moving from all high-quality to top-only predictions, this was not statistically significant, and the overall agreement between Hi-C and iPHoP remained stable. This suggests that host assignments are robust even when considering near-top predictions or multiple high quality predictions.

We have added Figure S8, add the methods used (lines 278-285), and posted the analysis scripts on GitHub at <https://github.com/100dru/SynCom_Hi-C>. Further, we have added a brief description of this analysis in the manuscript (lines 602-606):

“These results, which were robust to comparison strategies used (see Materials and Methods), suggest that while high-confidence computational predictions are largely consistent with Hi-C associations at broader taxonomic levels, they diverge at finer scales, particularly at the genus and species levels (**S8 Fig)**.”

**S8 Fig.** **Comparison of host predictions from iPHoP and Hi-C across three confidence thresholds, by habitat.** Bar plots show the agreement between host predictions from iPHoP and Hi-C across three habitats (palsa, bog, and fen). We used three different types of prediction filtering. Left column: All high-quality predictions (Hi-C Z-scores ≥ 0.5; iPHoP scores ≥ 90). Middle column: Near-top predictions (Hi-C Z-scores ≥ 0.5, all predictions with scores within 20% of the top score; iPHoP scores ≥ 90, all predictions with scores ±2 from top score). Right column: Top predictions (Hi-C Z-scores ≥ 0.5; iPHoP scores ≥ 90; only the top predictions, with multiple predictions if scores tied).

Results

24. Line 310: What was the cutoff threshold value for separating the signal from noise?

Response: Thank you for your question. After we performed read mapping, we normalized the read counts into contact scores, using a binomial regression-based analysis tool (MetaCC) that uses genome length, number of restriction sites and genome coverage to remove systemic biases. At this initial stage of the analysis (as referred to in the sentence in question), no cutoff was applied. Later in the study, we converted these contact scores into Z-scores to determine a statistically grounded threshold value for separating the signal from noise. We have amended the text for clarity (lines 332-333):

“SynCom-1 analyses revealed both expected and unexpected phage-host linkages, (prior to any data filtering).”

25. Provided three of the SynCom bacterial genomes represent the same species, their genomes might be very similar, and particular genomic fragments might be identical between the three. Did the authors check if such fragments exist? If yes, they could have contributed to the arise of false-positives. The same question applies to the SynCom phages.

Response: When designing the synthetic community, we specifically selected bacteria and phage isolates with known interactions (genomes listed in Table S1) that are as phylogenetically distant as possible within the Sullivan laboratory collection, based on average nucleotide identity (ANI).

To address your comment, we performed nucleotide-to-nucleotide BLAST alignments among both bacterial and phage genomes. These analyses revealed that the bacterial (and phage) genomes share fewer than 100 base pairs 100% identity. In addition, no significant alignments between bacteria and phage genomes. Thus, it is unlikely that ambiguous or identical genomic fragments contribute significantly to false positives in our dataset.

26. In the view of the comment above and overall based on the principles of the methodology, it appears appropriate to not even count the low-level contacts as linkages.

Response: Thank you for this observation. We agree that low-level contacts are suspicious and are likely to represent noise rather than true biological contacts. In this study, we incorporated Z-score filtering specifically to exclude such low-level contacts in a systematic and reproducible way across samples. This allows us to retain only statistically significant linkages while minimizing the inclusion of spurious low-level interactions.

27. Regarding the "unexpected linkages". Were the phage-bacteria in this experiment checked via an alternative approach (e.g. qPCR, microscopy)? If not, could there be a possibility that phages manifested an extended host range in the specific artificial community and conditions?

Response: Thank you for your question. The phages used in this study have been well characterized in the Sullivan lab using multiple methods, including plaque assays, lysis curves, and adsorption experiments, to define their host ranges. Prior to constructing SynComs-1, 2, and 3, we confirmed these host ranges under conditions matching those used in the study.

In addition, we have used these phage-bacteria combinations in similar SynCom setups previously, without observing evidence of extension of their host ranges. The consistent behavior of these phages across multiple controlled settings strongly suggests the unexpected linkages are not due to expanded host ranges. Thus, we do not think it is possible that the phages manifested an extend host range.

28. Fig. 2A: It might make sense to reduce the extent of the point jitter along the Y axis, as some points start approaching the points of another taxon.

Response: Thank you for your suggestion. We edited the Figure 2A (see below) to have less jitter.

29. Fig. 2B: Based on the description of the fills, the figure leaves an impression that the "Raw linkages" are way inferior in terms of the number of FP linkages. However, no threshold had been applied, unlike the case of Z-score. Would not it be a fair visual comparison if an optimal threshold were selected for "raw linkages" (based on the analysis of AUC, F1 score, G-means or other metric) and the respective plots were binarized akin to "Filtered linkages"?

Response: Thank you for your feedback. We agree that applying a threshold to the raw contact scores and comparing them in a binary fashion, similar to the Z-score filtered linkages, would make for a more direct visual comparison. However, we chose not to apply a fixed threshold to the raw linkages because contact score distributions vary substantially across technical replicates. This variability makes it difficult to define a single, globally applicable threshold without introducing bias.

That said, we did assess the discriminatory power by performing a Receiver Operating Characteristic (ROC) curve using raw contact scores for SynCom-1, which yielded an AUC of 0.71. This analysis is referenced in the main text (lines 369-376) and shown in Figure S3A. We believe this provides a fair evaluation of how well the raw scores perform without imposing an arbitrary threshold.

30. Line 326: Typo: "HI-c"

Response: Thank you for catching the error. We have fixed this typo (line 351).

31. Line 354: "most correct and incorrect linkages had Z-scores above or below 0, respectively" - Not sure if this phrase is informative, provided linkages belong to either correct or incorrect classes, no surprise their scores are non-zero.

Response: Thank you for catching this unclear phrasing. We revised the sentence for clarity (lines 379-380):

“Transforming SynCom-1 contact scores to Z-scores revealed that most correct linkages had Z-scores above 0, and this transformation improved the AUC to 0.93.”

32. Line 357: "Looking at the ROC curve further (S3 Fig), we selected a conversative threshold of 0.5 since it yielded the highest specificity and sensitivity across replicates." -- It is not clear how the value 0.5 was obtained from the shown curves. Overall, while visual inspection is valid for initial assessment, the analytical definition requires strictness: which criterion was used to select this specific threshold as optimal? (e.g. the one that optimizes F1 score, or G-means, or other metric). It's not possible to get the highest specificity and sensitivity simultaneously.

Response: Thank you for your comment. We have corrected the sentence (line 382-383): “…we selected a conversative threshold of 0.5 since it yielded the highest specificity across replicates.” We also appreciate the opportunity to clarify our rationale.

Ideally, threshold selection would involve systematically evaluating a range of Z-score cut-offs (e.g., 0.2 to 1.0) and comparing their performance using Receiver Operating Characteristic (ROC) analysis. In our study, we performed this analysis and found that the threshold maximizing Youden’s J statistic (i.e., optimizing the sum of sensitivity and specificity) was approximately 0.21. However, as shown in the revised Figure S3B (below), increasing the Z-score threshold consistently improves specificity at the cost of sensitivity, highlighting the inherent trade-off. We selected 0.5 (green) as a more conservative threshold that, while not maximizing Youden’s J, offers a balanced compromise.

**Figure S3B. Trade-off between sensitivity and specificity across Z-score thresholds for host-virus interaction predictions**. The plot illustrates the relationship between sensitivity and specificity for six different Z-score thresholds used to filter host-virus interactions. Each line represents a distinct threshold: 0 (red), 0.1 (orange), 0.25 (olive), 0.5 (green), 0.75 (cyan), and 1 (blue). The x-axis shows specificity, and the y-axis shows sensitivity.

33. Fig. S3: How were the data from 3 replicates summarized to get a single AUC? Please show the variability across the replicates, as well as the variability of the optimal threshold.

Response: Thank you for your question. We agree that showing the variability among replicates adds value to the interpretation of model performance. Due to the limited number of virus-host interaction predictions within each replicate of SynCom-1, it was not feasible to generate smooth or interpretable ROC curves for individual replicates. To address this, we pooled predictions from the three replicates and treated each prediction as an independent data point. This allowed us to compute a single AUC value that reflects the overall performance of the model across the entire dataset.

However, we acknowledge the importance of showing variability. In response, we added a table to the **Supporting Information** file including the AUC values obtained from each replicate with the caveat that the ROC curves did not have enough data so these numbers might be unreliable.

*Area Under the Curve for SynCom-1, SynCom-2 and SynCom-3 replicates*

| **Sample** | **AUC** |
| --- | --- |
| SynCom-1 replicate 1 | 0.9464286 |
| SynCom-1 replicate 2 | 0.9627329 |
| SynCom-1 replicate 3 | 0.8739496 |
| SynCom-2 replicate 1 | 0.8333333 |
| SynCom-2 replicate 2 | 0.8000000 |
| SynCom-2 replicate 3 | 1.0000000 |
| SynCom-3 replicate 1 | 0.8333333 |
| SynCom-3 replicate 2 | 1.0000000 |

34. Line 454: For the SynCom-2 and -3, please show that the optimal threshold previously obtained using a single community (SynCom-1) is still optimal (or at least performs well) - e.g. via an AUC analysis. Could it be the case that it's not optimal anymore suggesting lack of generalization and a need to compute an optimal threshold in a alternative way for each new sample?

Response: Thank you for your question. We agree that evaluating the performance of the SynCom-1 derived threshold using ROC curves for SynCom-2 and SynCom-3 would enhance our study. However, due to limited data points within each replicate, generating smooth ROC curves was not feasible, as noted in the previous comment (#34). As a compromise, we have added the individual AUC values for SynCom-2 and SynCom-3 replicates in the supporting information, accompanied by a cautionary note regarding the limited data (see previous comment).

**Reviewer #3** (Katherine McMahon):

Review of PBIOLOGY-D-25-01349_R1

Summary

This manuscript is an elegant effort to benchmark the approach of using the Hi-C method to infer virus-host pairs in multi-species assemblages. The authors used pure cultures of bacteria combined into synthetic communities, along with a collection of phage, to determine how well the Hi-C method recapitulates the experimentally validated ability of each phage to enter (infect?) each host. They examined the influence of multiplicity of infection on Hi-C sensitivity and specificity, evaluated several steps in the experimental protocol (both wet-lab and data-processing/normalization), and compared how Hi-C based host-virus predictions performed relative to bioinformatics approaches. Finally, they applied what they learned to environmental samples. Overall, the authors determine that the Hi-C approach is promising but should be used in combination with bioinformatics approaches, and with attention to detection limits and internal controls (e.g. spiked synthetic communities).

The manuscript is well-written, clear and accessible to read, and comprehensive in its description of the experimental design and findings. I'm sure the authors had hoped for more definitive results (and better performance of the Hi-C), but it is a solid contribution with insight that other researchers hoping to use this method in environmental samples, will greatly appreciate.

I have only a few suggestions to improve the manuscript.

Response: We thank the reviewer for their thoughtful and encouraging feedback. We appreciate your recognition of the clarity and comprehensiveness of our study. We agree that the benchmark provided here offers valuable insights and hope it will serve as a useful reference for future studies employing Hi-C for virus-host linkage analyses.

Major comments

35. The experiments conducted with varying MOI held the host abundance constant at 10^7 CFU/mL. As I understand it, this is 100x higher than typical seawater and 10x higher than freshwater (and hard to compare to soil/sediment/feces of course). I would like to have the authors add some speculation about how the HOST concentration would impact the results (beyond DNA yield), while holding the MOI constant. I'm not asking for more experiments, just a few sentences identifying this as a concern.

Response: Thank you for feedback. We added the following sentences to the main text (lines 500-503):

“Additionally, although this study does not explore variations in host concentration, we hypothesize that lowering host abundance while maintaining a constant MOI would result in fewer overall infected cells and altered spatial dynamics between viruses and hosts. Together, these factors could reduce the number and diversity of virus-host linkages detectable by Hi-C.”

36. Towards the end of the manuscript I found myself wondering whether the comparisons between Hi-C-predicted and bioinformatically-predicted was skewed in any way towards particular taxonomic groups. This would be an easy analysis to do, and would satisfy other readers with similar curiosity.

Response: Thank you for your insightful comment. We agree that taxonomy-focused analyses would be valuable additional analyses to do and appreciate the suggestion.

To address this, we compared the number of viruses assigned to each bacterial phylum by each method and performed a linear regression analysis. As shown in the new Figure S7B (below), there was a strong correlation between the two approaches (R²=0.852, *p* = 9.8 × 10⁻¹⁰), indicating that overall, at the phylum level, predictions are not systematically skewed toward specific groups.

However, three outliers were notable with two (Pseudomonadota and Actinomycetota) above the line indicating more virus-host linkages predicted by Hi-C than by iPHoP, and one (Acidobacteriota) slightly below the line, suggesting the opposite. These differences may reflect method-specific biases or biological characteristics of the taxa involved. For example, the elevated iPHoP predictions for Acidobacteriota likely stem from a high proportion (52%) of alignment-based matches, which rely on reference databases. In contrast, Pseudomonadota and Actinomycetota had lower proportions of alignment-based matches at 27% and 41%, respectively. The higher number of Hi-C-based linkages (like those associated with Pseudomonadota and Actinomycetota) may reflect a prevalence of lytic infections, which Hi-C is well-suited to detect due to its ability to capture physical interactions during active infection. Nonetheless, the overall strong correlation supports the robustness and consistency of our comparative framework.

We have added a summary of this analysis to the Results section (lines 617-620, see below) and included the plot in Figure S7B:

“Nonetheless, when comparing virus–host linkages aggregated across phyla, iPHoP and Hi-C methods showed strong overall agreement (R² = 0.852, p = 9.8 × 10⁻¹⁰; S7B Fig), suggesting that while taxon-specific biases exist, they do not preclude high-level concordance between approaches.”

**Figure S7 B.** **Taxonomic consistency between Hi-C and bioinformatic host-virus predictions.** Scatter plot showing the relationship between the number of virus-host associations predicted per bacterial phylum by the bioinformatic method (iPHoP; x-axis) and the Hi-C method (y-axis). Each point represents a distinct phylum (with three dots labelled by phylum name as an example). A linear regression reveals a strong positive correlation between the two methods (R² = 0.852, p = 9.8 × 10⁻¹⁰), indicating that both approaches produce broadly similar taxonomic distributions of predicted hosts.

Minor comments (intended to increase clarity and ease of interpretation):

37. Line 164: Pet peeve: this number has far too many significant figures. Use 4 x 10^7 instead.

Response: Thank you for your comment. We have adjusted the number to 4 x 10^7^ (line 160).

38. Line 285-286. In my first read-through, the term "positive and negative interactions" was confusing (mostly the latter). I'm assuming you mean a host-phage pair that CAN interact (positive) and a pair that CANNOT interact (negative). The terms do make sense in the context of "false negative", so I don't know why it's confusing (but it is)! Consider re-wording to clarify, or at least explain?

Response: Thank you for your suggestion. Your understanding is correct – a positive interaction refers to a host–phage pair in which the phage can successfully infect the host, while a negative interaction refers to a pair where infection does not occur. To improve clarity and avoid potential confusion, we have revised the language (lines 303-306):

“This SynCom mix of bacteria and phage strains were chosen to examine how varying infection dynamics influenced Hi-C performance, including infective and non-infective phages relative to the host community, and these quantitatively defined interactions were then used as the basis for Hi-C performance assessments.”

39. Line 300: I think you are referring to Figure 1B.

Response: Thank you for catching this error. We have revised the text to refer to Figure 1B (line 319).

40. Line 314: The term "negative interactions" confused me (see comment about line 285-286 above).

Response: Thank you for the comment. We changed the wording to enhance clarity (lines 335-336):

“Unexpected linkages were for phage-bacteria pairs not known to infect that we included to represent negative controls.”

41. Lines 342-351: I suggest moving this paragraph to the supplementary material. I realize that it fits in the narrative of "first we tried this, then we tried that", but it doesn't add much to the main text since it's not mentioned in a main figure.

Response: Thank you for your suggestion. We appreciate your insights regarding the quality of the story. We also debated whether this paragraph should appear in the main text or be moved to the Methods or Supplementary Materials.However, after careful consideration, we chose to retain it in the main text because we believe it provides valuable guidance for future users of Hi-C. The current literature offers limited discussion on the application of ROC curves in this context, with many approaches for noise reduction appearing somewhat arbitrary (e.g. requiring a minimum of two virus-host linkages (9,10)) which is further showcased in S8 Table. We believe this clarification contributes to the transparency and reproducibility of our approach.

42. Line 443: The host concentration should be in units of CFU/mL, right?

Response: Thank you for the correction. You are correct – host concentration should be reported in CFU/mL rather than PFU/mL. We have corrected this (line 470) to reflect the appropriate units.

43. Line 471: How does the detection limit nominally depend on host cell abundance? It seems like the MOI would be the more important metric, but maybe I am mis-interpreting.

Response: Thank you for the comment. It was an error. We have edited the main text to replace detection limit with MOI (line 498-499).

44. Line 507: I think there are two words missing: "..varied substantially by habitat, WITH bog BEING the highest…"

Response: Thank you for catching this error. This has been revised in the text (line 544).

45. Figure 1: Panel B, for the hosts, I think this should be CFU/mL not PFU/mL? I appreciated this figure, since it helped a lot to understand the setup.

Response: Thank you for catching this error. You are correct, the host concentrations should be reported in CFU/mL rather than PFU/mL. We have revised Figure 1B to show CFU/mL. We are pleased to hear that the figure helped clarify experimental set up.

46. Figure 2A: What is the meaning behind the normalized linkage score? Is it useful to compare across replicates, or is it only appropriate to compare within a replicate? I assume the latter, since that would motivate using the z-score. For people not familiar with Hi-C, it would help to explain this in the legend, tho. Also, what is the difference between a contact score and a linkage score? I suspect they are synonyms, so stick with one please.

Response: Thank you for your comment. We acknowledge that the terms "linkage score," and "contact score" were used interchangeably in the original text, which may have caused confusion. We have revised the manuscript to ensure consistent and accurate use of terminology throughout. For clarity, we added a paragraph in the Methods sections explaining the definitions of contact score and other relevant terms. Additionally, we have updated the Figure 2 legend to include the definition of the contact score.

In the Methods (lines 216-222), we added:

“For clarity, after reference mapping, we obtain read counts for each contig (associated with a MAG or virus). MetaCC then converts these read counts into *contact scores*, by normalizing them based on the number of restriction sites, genome length, and sequencing coverage. A contact score represents the Hi-C interaction metric between a virus and a host. We refer to any virus–host pair with a contact score as a *linkage*. Throughout the manuscript, we distinguish between *raw linkages*, which include all virus–host pairs with a contact score, and *filtered linkages*, which are those retained after applying a Z-score-based filtering step.”

In the Figure 2 legend (lines 344-348), we added this definition:

“The contact score represents the number of Hi-C linkages between a virus and a host genome, normalized for the number of restriction sites, genome length, and coverage. Z-scores were calculated from the contact scores within each sample to enable comparison across samples.”

**References:**

1.	Han Z, Wei G. Computational tools for Hi-C data analysis. Quant Biol. 2017 Sept 1;5(3):215–25.

2.	Efficient and easy‐to‐use capturing three‐dimensional metagenome interactions with GutHi‐C - Lu - 2024 - iMeta - Wiley Online Library [Internet]. [cited 2025 Sept 16]. Available from: https://onlinelibrary.wiley.com/doi/full/10.1002/imt2.227

3.	Braid MD, Daniels LM, Kitts CL. Removal of PCR inhibitors from soil DNA by chemical flocculation. J Microbiol Methods. 2003 Mar 1;52(3):389–93.

4.	Zimmerman BD, Korajkic A, Brinkman NE, Grimm AC, Ashbolt NJ, Garland JL. A Spike Cocktail Approach to Improve Microbial Performance Monitoring for Water Reuse. Water Environ Res. 2016;88(9):824–37.

5.	Koyuncu S, Andersson MG, Häggblom P. Accuracy and Sensitivity of Commercial PCR-Based Methods for Detection of Salmonella enterica in Feed. Appl Environ Microbiol. 2010 May;76(9):2815–22.

6.	Brinch UC, Ekelund F, Jacobsen CS. Method for Spiking Soil Samples with Organic Compounds. Appl Environ Microbiol. 2002 Apr;68(4):1808–16.

7.	Fu Z, Ai J, Zhang H, Cui P, Xu T, Zhang Y, et al. Pathogen quantitative efficacy of different spike-in internal controls and clinical application in central nervous system infection with metagenomic sequencing. Microbiol Spectr. 2023 Nov 20;11(6):e01139-23.

8.	Shamash M, Sinha A, Maurice CF. Improving gut virome comparisons using predicted phage host information. mSystems. 2025 Apr 8;10(5):e01364-24.

9.	Wu R, Davison MR, Nelson WC, Smith ML, Lipton MS, Jansson JK, et al. Hi-C metagenome sequencing reveals soil phage–host interactions. Nat Commun. 2023 Nov 23;14(1):7666.

10.	Cheng Z, Li X, Palomo A, Yang Q, Han L, Wu Z, et al. Virus impacted community adaptation in oligotrophic groundwater environment revealed by Hi-C coupled metagenomic and viromic study. J Hazard Mater. 2023 Sept 15;458:131944.
